# Genome assembly of Genji firefly (*Nipponoluciola cruciata*) reveals novel luciferase-like luminescent proteins without peroxisome targeting signal

**DOI:** 10.1093/dnares/dsae006

**Published:** 2024-03-18

**Authors:** Kentaro Fukuta, Dai-ichiro Kato, Juri Maeda, Atsuhiro Tsuruta, Hirobumi Suzuki, Yukio Nagano, Hisao Tsukamoto, Kazuki Niwa, Makoto Terauchi, Atsushi Toyoda, Asao Fujiyama, Hideki Noguchi

**Affiliations:** Center for Genome Informatics, Joint Support-Center for Data Science Research, Research Organization of Information and Systems, Mishima, Shizuoka 411-8540, Japan; Data Analysis Division, Advanced Genomics Center, National Institute of Genetics, Mishima, Shizuoka 411-8540, Japan; Department of Science, Graduate School of Science and Engineering, Kagoshima University, Kagoshima 890-0065, Japan; Department of Science, Graduate School of Science and Engineering, Kagoshima University, Kagoshima 890-0065, Japan; Department of Science, Graduate School of Science and Engineering, Kagoshima University, Kagoshima 890-0065, Japan; Japan Fireflies Society, Hino, Tokyo 191-0016, Japan; Analytical Research Center for Experimental Sciences, Saga University, Saga 840-8502, Japan; Department of Biology, Graduate School of Science, Kobe University, Kobe 657-8501, Japan; Advanced Quantum Measurement Group, Research Institute for Physical Measurement, National Metrology Institute of Japan, National Institute of Advanced Industrial Science and Technology (AIST), Tsukuba 305-8563, Japan; Center for Genome Informatics, Joint Support-Center for Data Science Research, Research Organization of Information and Systems, Mishima, Shizuoka 411-8540, Japan; Data Analysis Division, Advanced Genomics Center, National Institute of Genetics, Mishima, Shizuoka 411-8540, Japan; Comparative Genomics Laboratory, Department of Genomics and Evolutionary Biology, National Institute of Genetics, Mishima, Shizuoka 411-8540, Japan; Sequencing Division, Advanced Genomics Center, National Institute of Genetics, Mishima, Shizuoka 411-8540, Japan; Data Analysis Division, Advanced Genomics Center, National Institute of Genetics, Mishima, Shizuoka 411-8540, Japan; Comparative Genomics Laboratory, Department of Genomics and Evolutionary Biology, National Institute of Genetics, Mishima, Shizuoka 411-8540, Japan; Center for Genome Informatics, Joint Support-Center for Data Science Research, Research Organization of Information and Systems, Mishima, Shizuoka 411-8540, Japan; Data Analysis Division, Advanced Genomics Center, National Institute of Genetics, Mishima, Shizuoka 411-8540, Japan

**Keywords:** firefly, whole genome, firefly luciferase, thioesterase, opsin

## Abstract

The Genji firefly, *Nipponoluciola cruciata*, is an aquatic firefly endemic to Japan, inhabiting a wide area of the Japanese archipelago. The luminescence of fireflies is a scientifically interesting phenomenon, and many studies have evaluated this species in Japan. In this study, we sequenced the whole genome of male *N. cruciata* and constructed a high-quality genome assembly of 662 Mb with a BUSCO completeness of 99.1% in the genome mode. Using the detected set of 15,169 protein-coding genes, the genomic structures and genetic background of luminescence-related genes were also investigated. We found four new firefly luciferase-like genes in the genome. The highest bioluminescent activity was observed for LLa2, which originated from ancestral PDGY, a mitochondrial acyl-CoA synthetase. A thioesterase candidate, NcruACOT1, which is involved in d-luciferin biosynthesis, was expressed in the lantern. Two opsins were also detected and the absorption wavelength of the UV-type opsin candidate shifted from UV to blue. These findings provide an important resource for unravelling the adaptive evolution of fireflies in terms of luminescence and vision.

## 1. Introduction

The light of fireflies has an allure that attracts attention around the world. In Japan, fireflies have fascinated people since ancient times, and many descriptions of fireflies can be found in ancient books. For example, fireflies are documented in the Man’yōshū, a collection of Japanese poems (waka) compiled about 1,300 yrs ago. About 50 species of fireflies are known in Japan. One of the most well-known firefly species is Genji firefly, *Nipponoluciola cruciata* (formerly *Luciola cruciata*). This firefly is endemic to Japan and is distributed throughout the three main islands (Honshu, Shikoku, and Kyushu). Two ecological types have been recognized based on the synchronous flash behaviour of males (Supplementary Fig. S1).^1–3^ The habitation boundary corresponds to a great rupture zone termed the Fossa Magna, which divides Honshu island into eastern (East-Honshu) and western (West-Honshu) areas.^1^ Previous genetic studies of this species have been primarily based on mitochondrial sequences^4,5^ or partial genome sequences.^1^

The bioluminescence of fireflies is generated by the catalytic activity of the luciferase enzyme in the luminous organ, the lantern.^6^ This enzyme belongs to the acyl-CoA synthetase (ACS) gene superfamily. ACS genes have expanded through gene duplication events in firefly lineages,^7,8^ and some are thought to have acquired luminescent activity. While most ACSs are involved in fatty acid β-oxidation in mitochondria, those with peroxisome targeting signal 1 (PTS1) work in peroxisomes. All known firefly luciferases are derived from a peroxisomal ACS (PACS) and have the PTS1 signal.^7^ ACSs without PTS1 have also expanded in the firefly genomes; however, most of these have not been examined for luminescent activity.

The substrate of the firefly bioluminescence reaction is d-luciferin. This compound has an asymmetric carbon atom in the molecule, and its chirality is crucial because another enantiomer, l-luciferin, is a potent competitive inhibitor of the light-emitting reaction.^9,10^ Detailed analyses of chirality have suggested that l-luciferin is the biosynthetic precursor of d-luciferin in fireflies.^11,12^ Fireflies effectively produce d-luciferin from the l-form enantiomer by a chiral inversion process through the intermediate luciferyl coenzyme A (luciferyl-CoA).^4^ The chiral inversion mechanism is a deracemization process involving three reactions: enantioselective thioesterification, epimerization, and thioester hydrolysis. In the process, the l-luciferyl-CoA intermediate is initially produced from l-luciferin by l-enantioselective thioesterification via luciferase. l-Luciferyl-CoA rapidly epimerizes non-enzymatically through enol formation, and d-luciferin is formed by hydrolysis of the epimerized intermediate d-luciferyl-CoA by thioesterase. Recently, the acyl-CoA thioesterase *ACOT1* of *Abscondita terminalis* (Lampyridae) was identified as a strong candidate thioesterase responsible for the conversion of l-luciferin to d-luciferin.^8^

Visual detection of the emitted light is important for sexual communication in fireflies. Although detailed analyses of the compound eye of fireflies have been performed from anatomical^13^ and neurosensory^14^ perspectives, the molecular basis of their visual system is not fully understood. Two types of opsin genes have been isolated from adult *N. cruciata*; based on their sequence similarities, these genes were predicted to encode a long-wavelength-sensitive opsin (LW-opsin) and an ultraviolet-sensitive opsin (UV-opsin), and the LW-opsin is thought to be responsible for the ability to discriminate the bioluminescence signal.^13^ The absorption wavelengths of various fireflies have been measured using the electroretinogram (ERG),^14^ which measures the electrical responses of various cell types in the retina. In contrast, the spectral absorption property of the photopigment constructed by the opsin with retinal (chromophore) has not been directly determined.

In this study, we sequenced the entire genome of male *N. cruciata* and investigated the genomic structure and the genetic background of luminescence-related genes, such as luciferase-like ACSs, thioesterases, and opsins. First, we focussed on luciferase-like ACSs without PTS1 and investigated their luminescent activities and structures. Second, we identified *ACOT* genes, including *ACOT1*, in the *N. cruciata* genome and performed a comparative analysis with those in the related species. Finally, we performed a phylogenetic analysis of opsin genes and measured the absorption wavelengths of *N. cruciata* opsins.

## 2. Materials and methods

### 2.1. Sample collection, library preparation, and sequencing

Adult male *N. cruciata*, categorized as a West-Honshu type specimen, was purchased from Yamato no Kuni Kawaguchi, a firefly farmer in Yamazoe, Nara, Japan. The entire body was homogenized by freeze-grinding using a ball mill, excluding the wings and tail. According to the manufacturer’s instructions, genomic DNA was extracted using the Blood & Cell Culture DNA Mini Kit (Qiagen, Hilden, Germany), and the concentration was assessed using the QuantiFluor ONE dsDNA System (Promega, Madison, WI, USA). The extracted DNA was sheared using the M220 Focused-ultrasonicator (Covaris, Woburn, MA, USA). Paired-end libraries were prepared using the TruSeq DNA PCR-Free Library Prep Kit (Illumina, San Diego, CA, USA) and were purified using Agencourt AMPure XP (Beckman Coulter, Brea, CA, USA). Mate-pair libraries were prepared using the Nextera Mate Pair Sample Preparation Kit (Illumina). The DNA concentrations were measured using the Agilent 2100 Bioanalyzer (Agilent Technologies, Santa Clara, CA, USA), and all libraries were sequenced on the Illumina HiSeq 2500 platform.

Total RNA was extracted from the tail including luminescent organs of the firefly sample using ISOGEN (Nippon Gene, Tokyo, Japan), and an RNA sequencing (RNA-seq) library was prepared using the TruSeq Stranded mRNA Library Prep Kit (Illumina). The cDNA sample was used following 15 cycles of PCR amplification without size selection. The library was sequenced on the Illumina HiSeq 2000 platform.

### 2.2. Genome assembly and annotation

The *N. cruciata* genome was assembled using Platanus-allee v2.0.2^15^ with default parameters. The paired-end reads were used to construct contigs, and both the paired-end and mate-pair reads were used for scaffolding and gap-closing. In advance, the adaptor sequences in the short reads were trimmed using Fastp v0.23.2,^16^ and all reads mapped to the mitochondrial genome (NC_022472.1)^5^ with an edit distance less than 4 by BWA-mem2 v2.2.1^17^ were also removed. Short scaffolds (i.e. <1 kb) were removed, and redundant sequences were also removed using purge_dups v1.2.5^18^ to construct the final genome assembly, which was designated as NipCru1. The paired-end reads were remapped to NipCru1 and used to assess the genome assembly quality and calculate the heterozygosity. BWA-mem2 was used with the default parameters for the remapping. Duplicated reads were removed using MarkDuplicates of the GATK pipeline (v4.0.8.1),^19^ and only reads that mapped uniquely to the genome with > 92% identity were retained. The variant calling was performed using HaplotypeCaller of the GATK pipeline with the default parameters. Genomic regions with half to twice the average mapping depth and with reads mapped to both strands were included, and the 10 bp ranges before and after indels were excluded from the analysis. *K*-mer (*k* = 35) frequencies in the paired-end reads were also examined using jellyfish v2.2.10^20^ and GenomeScope v1.0^21^ to estimate a genome size and heterozygosity.

A library of repetitive elements for NipCru1 was constructed using RepeatModeler v2.0.2 (https://www.repeatmasker.org/RepeatModeler/), and repeat regions were masked using RepeatMasker v4.1.0 (https://www.repeatmasker.org) with the constructed library. The protein-coding genes were identified on the masked genome sequence based on the RNA-seq alignment, homology search with known genes, and *ab initio* gene prediction. The RNA-seq reads were assembled using Trinity v2.6.6,^22^ and the assembled transcripts were aligned to the genome using the PASA pipeline v2.4.1.^23^ Known protein sequences were obtained from UniRef90 rel.202006^24^ and first aligned to the genome using MMseqs2 v13.45111.^25^ Subsequently, Spaln v2.4.01^26^ with the parameter ‘-T InsectCo’ was used for the spliced alignment of candidates to predict precise gene structures. *Ab initio* gene prediction was performed using Augustus v1.2.3^27^ under the Funannotate pipeline v1.8.7^28^ with the RNA-seq raw reads, assembly, and protein alignments as training data. The final gene set was constructed as follows. For each gene locus (genomic region), a gene model having the longest coding sequence among gene candidates from the RNA-seq- and homology-based predictions was selected as representative. Then, all candidates that shared some exons with the representative model were selected as transcript variants. Finally, when *ab initio* models categorized as ‘high confidence’ were in the intergenic regions, they were merged into the gene set. Gene functions were annotated using InterProScan v5.55-88.0.^29^ Gene expression levels in the luminescent organ were calculated as TPM (Transcripts Per Kilobase Million) using HISAT2 v2.2.1^30^ and StringTie v2.2.1.^31^ The completeness of the gene set was evaluated by BUSCO v5.2.2^32^ with the ‘insecta_odb10’ database in the ‘proteins mode’. BUSCO was performed using only the longest isoforms as representatives to compare BUSCO scores with other fireflies having no isoform annotations. tRNAs and rRNAs were annotated using tRNAscan-SE v1.3.1^33^ and RNAmmer v1.2,^34^ respectively.

### 2.3. Comparative analyses of luminous beetles

Comparative analyses of *N. cruciata* and five luminous beetles, *Aquatica lateralis* (formerly named *Luciola lateralis*), *Photinus pyralis*, *Ignelater luminosus*, *A. terminalis*, and *Lamprigera yunnana*, were performed using the following datasets. The genome assemblies of *Aq. lateralis* (Alat1.3), *P. pyralis* (Ppyr1.3), and *I. luminosus* (Ilumi1.2) were obtained from Fireflybase,^7^ and their official gene sets (AQULA_OGS1.0, PPYR_OGS1.1, and ILUMI_OGS1.2, respectively) were obtained from the GitHub repositories of eLife Sciences. The genome assemblies and annotations of *Ab. terminalis* (Ate, GCA_013368085.1)^8^ and *L. yunnana* (ASM1336807v1, GCA_013368075.1)^8^ were obtained from NCBI GenBank.

The phylogenetic relationships among these species were examined as follows. Orthogroups were determined using the reciprocal best-hit (RBH) workflow of MMseqs2 (easy-rbh), and multiple sequence alignments were constructed using Clustal Omega v1.2.4.^35^ The phylogenetic tree was inferred based on the maximum likelihood method using RAxML-NG v1.1.0^36^ with the ‘DAYHOFF+G4’ model, identified using ModelTest-NG v0.1.6.^37^ The divergence times were estimated using RelTime-ML implemented in MEGA11.^38^ Phylogenetic trees based on specific genes, such as firefly luciferases, thioesterases, and opsins, were also constructed based on the maximum likelihood method using RAxML-NG with the ‘LG+G4’ model for luciferases and opsins and the ‘PMB+G4’ model for thioesterases.

To calculate the *K*_a_/*K*_s_ ratios of acyl-CoA thioesterases (ACOTs), the ACOT proteins of *N. cruciata* were aligned with the orthologues of *Ab. terminalis* using Clustal Omega, then KaKs_Calculator 2.0^39^ was applied with default parameters.

### 2.4. Identification of luciferase-like proteins

All detected genes in NipCru1 were clustered using the gene clustering workflow of MMseqs2 (easy-cluster), setting ‘--min-seq-id 0.3’, and the luciferase-related ACS cluster containing *LUC1* was identified. The presence of PTS1 in the identified ACSs was estimated using the PTS1 predictor.^40^ Among luciferase-related ACSs, those that had the ‘Firefly-Luc-like’ motif (CDD^41^: cd05911) and were expressed in the lantern were specifically defined as luciferase-like (luc-like) proteins. 3D structural models of the luc-like proteins were calculated using ColabFold: AlphaFold2 (1 February 2022) using MMseqs2 with Advanced settings [msa_mode: MMseqs2 (UniRef+Environmental), model_type: AlphaFold2-ptm, pair_mode: unpaired, num_recycle: 3], without templates^42^ (https://colab.research.google.com/github/sokrypton/ColabFold/blob/main/AlphaFold2.ipynb).

### 2.5. Expression and purification of recombinant luc-like proteins

The cDNAs of the target sequences were synthesized (Fasmac, Kanagawa, Japan) with *Escherichia coli* codon usage optimization and ligated into the expression vector pCold-GST^43^ using the SLiCE method.^44^  *Escherichia coli* BL21(DE3) cells were transformed with a constructed expression vector, cultivated, and used for recombinant protein expression by a cold shock procedure. After overnight culture, cells were harvested and disrupted by sonication (20 kHz, 30 s × 10 times) in 50 mM potassium phosphate buffer (pH 7.0) containing 300 mM NaCl. After centrifugation (14,500 × *g* for 10 min, 4°C), the target protein was purified from the supernatant using TALON Metal Affinity Resin (Clontech, Mountain View, CA, USA) according to the manufacturer’s instructions. Active fractions were combined and dialysed overnight in 100 mM Tris–HCl buffer (pH 8.0). Protein concentrations were measured using Bio-Rad Protein Assay Dye Reagent (Bio-Rad, Hercules, CA, USA) with bovine serum albumin as a standard. The purified protein was stored at −30°C until use after adding glycerol to reach 10%.

### 2.6. Measurement of luminescence intensity and spectra

The luminescence intensity was measured using a luminometer CLX-101 (TOYOBO, Osaka, Japan). The luminescence reaction was initiated by injecting 40 µl of ATP (final conc. 1.6 mM) and MgCl_2_ (final conc. 3.2 mM) solution in 100 mM Tris–HCl buffer (pH 8.0) into 60 µl of a mixture of purified luc-like protein (total 500 ng protein) and d-luciferin (final conc. 400 µM). After mixing the light counts were integrated for 10 s.

The emission spectra were measured using an AB-1850 LumiFL-Spectrocapture (Atto, Tokyo, Japan) (slit width, 0.5 mm; spectral resolution, 0.5 nm) for 30 s with the bioluminescent solution in a 0.2 ml PCR tube (total 50 µl) containing purified luc-like protein (total 8 µg protein), d-luciferin (final conc. 400 µM), ATP (final conc. 1.6 mM), and MgCl_2_ (final conc. 3.2 mM) in 100 mM Tris–HCl buffer (pH 8.0). All spectra were corrected for the spectral sensitivity of the equipment and normalized.

### 2.7. Expression and purification of UV- and LW-opsin candidates in *N. cruciata*

The cDNAs of the target sequences were synthesized (Fasmac) with *E. coli* codon use optimization and inserted into the *Eco*RI/*Not*I site in the mammalian expression vector pMT using In-Fusion HD (Takara Bio, Kusatsu, Japan). At the C terminus, the amino acid sequence of the 1D4 tag (ETSQVAPA), which is a recognition sequence of the antibody 1D4, was added for purification using 1D4 antibody columns. Candidate opsins were transiently expressed in COS-1 cells (10 plates, 100 mm diameter), which were harvested for 8 h at 37°C after transfection and incubated for an additional 88 h at 30°C. The harvested cells were incubated with 11-cis-retinal overnight, and membrane proteins were solubilized with 1.25% DDM (n-Dodecyl-β-D-maltoside, Dojindo, Kumamoto, Japan), 20 mM HEPES (4-(2-hydroxyethyl)-1-piperazineethanesulfonic acid), 140 mM NaCl, 0.25% cholesterol hemisuccinate (Sigma-Aldrich, St. Louis, MO, USA), 25 mM Tris, and 10% glycerol (pH 7.0). The solubilized materials were mixed with 1D4-agarose overnight, and the mixture was transferred to Bio-Spin columns (Bio-Rad). The columns were washed with 0.05% DDM, 2 mM ATP, 1 M NaCl, 3 mM MgCl_2_, 0.01% cholesterol hemisuccinate, 1 mM Tris, and 10% glycerol in phosphate-buffered saline and subsequently washed with 0.05% DDM, 140 mM NaCl, 0.01% cholesterol hemisuccinate, 1 mM Tris, 10% glycerol, and 20 mM HEPES (pH 7.0) (buffer A). The 1D4-tagged pigments were eluted with buffer A containing 0.45 mg/ml 1D4 peptide (TETSQVAPA) (Toyobo, Osaka, Japan). After adding 11-cis-retinal, all procedures were conducted under 660 nm LED red light.

### 2.8. UV–visible spectroscopy and photoreaction of opsin candidates

The absorption spectra of the purified photopigments were recorded using a Shimadzu UV-2450 spectrophotometer (Kyoto, Japan). The samples were maintained at 10°C. The details are described in a previous study.^45^

## 3. Results and discussion

### 3.1. Genome assembly and annotation

We performed *de novo* genome assembly of *N. cruciata* using Illumina short reads (Supplementary Table S1 and Supplementary Fig. S2). The total size of the constructed genome assembly, designated as NipCru1, was 662 Mb and the scaffold N50 length was 48.3 Mb (Table 1 and Supplementary Table S2). NipCru1 had a very low GC (guanine-cytosine) content of 28.4% and the percentage of repetitive elements was 32.3% (Table 1). The paired-end reads were remapped to NipCru1 to check the genome assembly quality and 99.4% of the reads were successfully remapped (Supplementary Table S3). The heterozygosity of *N. cruciata* was calculated from the mapped reads (Supplementary Table S4). Variant calling was made using 82.9% of the uniquely mapped reads covering 91.1% of the genome. Approximately 6.2 million single nucleotide polymorphisms (SNPs) were found, and the heterozygosity of *N. cruciata* was estimated to be 1.0%. We identified 15,169 protein-coding genes comprising 23,021 transcripts in NipCru1. We also identified 113 tRNAs and 29 rRNAs. The BUSCO completeness with insecta_odb10 was 99.1% in the genome mode and 98.1% in the proteins mode (Table 1 and Supplementary Table S2), indicating the high quality of the NipCru1 genome assembly and annotation.

**Table 1.** Statistics of the firefly genome assemblies and annotations

Species	Reference	Genome size (bp)	GCcontent (%)	Repeats (%)	Gene loci	Transcripts	BUSCO^a^
*Nipponoluciola cruciata* (NipCru1)	This study	662,010,827	28.4	32.3	15,169	23,021	C: 98.1% [S: 97.4%, D: 0.7%], F: 0.5%, M: 1.4%
*Aquatica lateralis* (Alat1.3)	Fallon *et al*.^7^	908,530,830	25.0	19.8	14,284	14,284	C: 90.7% [S: 90.0%, D: 0.7%], F: 2.2%, M: 7.1%
*Abscondita terminalis* (Ate)	Zhang *et al*.^8^	499,652,588	31.4	35.5	20,439	20,439	C: 95.3% [S: 92.2%, D: 3.1%], F: 1.1%, M: 3.6%
*Lamprigera yunnana* (ASM1336807v1)	Zhang *et al*.^8^	1,052,929,944	34.1	66.6	19,438	19,438	C: 94.9% [S: 93.2%, D: 1.7%], F: 1.2%, M: 3.9%
*Photinus pyralis* (Ppyr1.3)	Fallon *et al*.^7^	471,511,253	36.4	42.6	15,773	15,773	C: 95.4% [S: 84.1%, D: 11.3%], F: 0.3%, M: 4.3%
*Ignelater luminosus* (Ilumi1.2)	Fallon *et al*.^7^	842,761,136	32.0	34.1	27,558	27,558	C: 94.5% [S: 92.2%, D: 2.3%], F: 2.0%, M: 3.5%

^a^BUSCO scores (proteins mode) against insecta_odb10 for C: complete, S: single, D: duplicate, F: fragmented, and M: missing.

### 3.2. Phylogenetic relationships of luminous beetles

**Figure 1.**
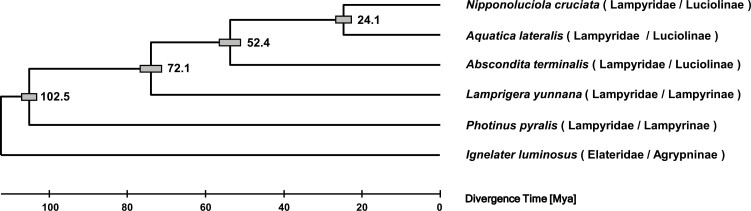
Phylogenetic tree of luminous beetles. The numbers on the tree indicate divergence times in million years based on the divergence time of lucioline and lampyrine (100–105 Mya). Grey boxes indicate the confidential intervals of the divergence times.

The maximum likelihood tree of the luminous beetles (five lampyrids and one elaterid) inferred from 4,649 orthogroups is shown in Fig. 1. *Nipponoluciola cruciata* and *Aq. lateralis* were most closely related, followed by *Ab. terminalis*. These species belong to Luciolinae. Two lampyrine species, *L. yunnana* and *P. pyralis*, were more distantly related. Finally, the elaterid beetle, *I. luminosus*, was located at the base of the tree. To estimate divergence times among the species, a divergence time estimate of 100–105 million yrs ago (Mya) between lucioline and lampyrine^7^ was used for time calibration. The divergence time between *N. cruciata* and *Aq. lateralis* was estimated to be ~24.1 Mya (confidential interval: 22.2–26.2 Mya). This value was almost the same as the divergence time (21 Mya) based on the mitochondrial cytochrome c oxidase subunit II (COII) gene.^46^ In this case, the molecular evolutionary rate of COII in a chrysomelid beetle was adopted.^47^

The Japanese archipelago began to form in the Eocene epoch (56–34 Mya) and separated from the Eurasian continent in the Miocene epoch (23–5.3 Mya) due to the movement of oceanic plates, resulting in the formation of the Japan Sea. The divergence time between *N. cruciata* and *Aq. lateralis* aligns with the formation of the Japan Sea. The two species in the genus *Nipponoluciola* are endemic to Japan, while the five species in *Aquatica* are distributed in East Asia. In particular, only *Aq. lateralis* inhabits wide areas from East Asia to East Siberia, including Japan. The divergence time deduced from the phylogenetic tree based on genome sequences has important implications for considering the roles of geological events and the speciation process in *N. cruciata*, *Aq. lateralis*, and closely related species in East Asia. The genome size of *Aq. lateralis* (909 Mb) was significantly larger than those of other lucioline species, *N. cruciata* (662 Mb) and *Ab. terminalis* (500 Mb). The difference in genome size between *N. cruciata* and *Aq. lateralis* is probably due to the genome expansion in *Aq. lateralis*. The underlying driving force of the expansion is unknown and needs to be clarified in future studies.

### 3.3. Luminescence and structural properties of novel luciferase-like proteins

We found 87 ACSs in the *N. cruciata* genome. A clustering analysis revealed 22 luciferase-related ACSs, including functional luciferases, LUC1 and LUC2 (Supplementary Table S5). Among them, ACSs that were expressed in the lantern (TPM > 10) and had a motif ‘Firefly-Luc-like’ (cd05911) were defined as luciferase-like (luc-like) proteins. Seven luc-like proteins (LLp1–4, LLa1–3) were identified as candidate novel luciferases. To examine luminescence properties, four kinds of luc-like proteins were selected: LLp2 and LLa2 were the first and second most highly expressed gene products in the lantern, and LLp1 and LLp3 were the most distal and most proximal relatives to LLp2 within the LLp clade (see Section 3.4).

**Figure 2.**
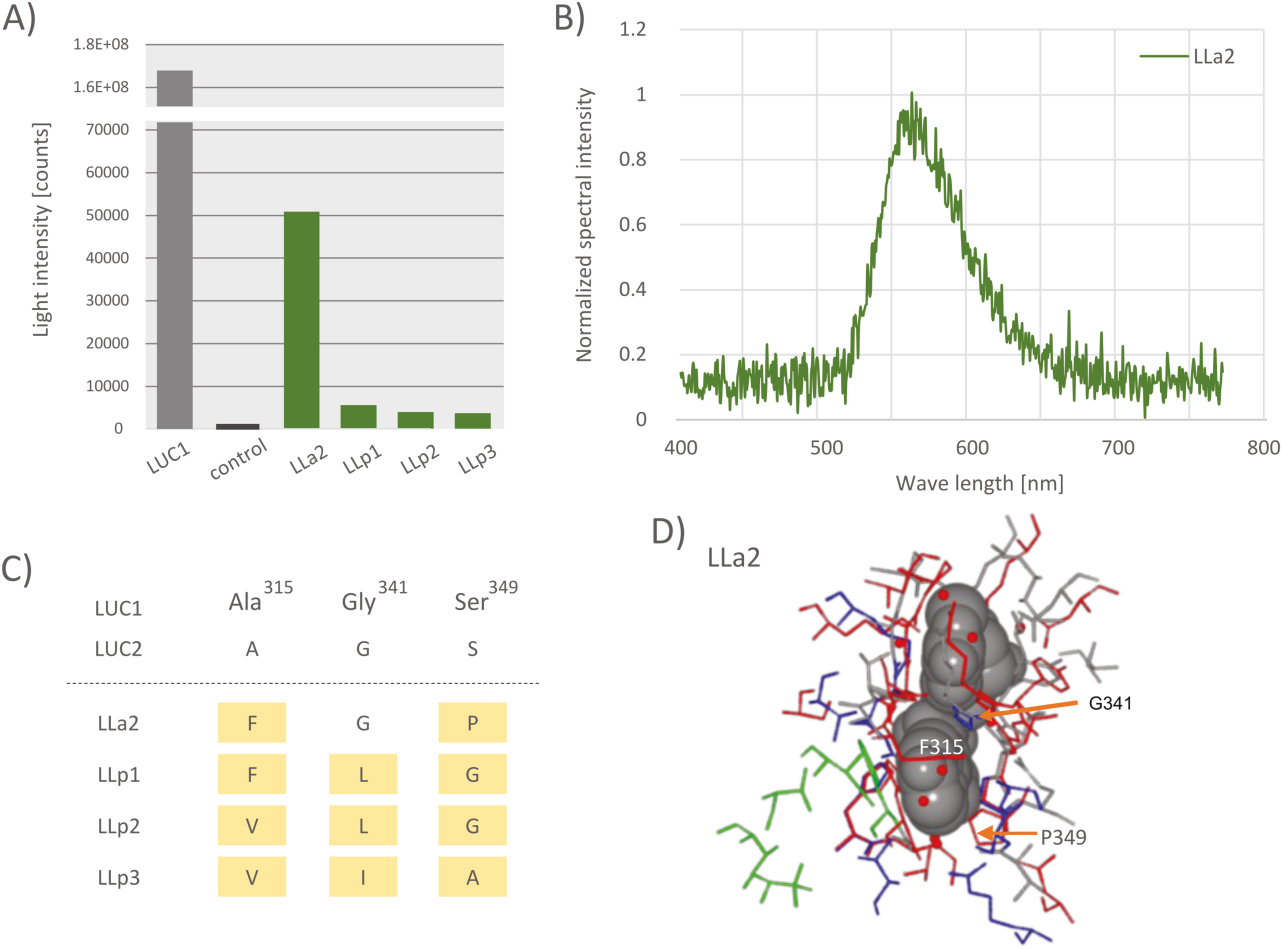
Bioluminescence properties and predicted protein structure of newly discovered four luciferase-like proteins, LLp1, LLp2, LLp3, and LLa2. (A) The luminescence intensity of luciferase-like proteins. (B) The emission spectrum of the bioluminescence reactions for LLa2 protein at pH 8.0. The spectrum was normalized at the maximum wavelength. (C) Comparison with luciferase-like proteins and LUC1 at extracted three amino acid residues discussed in the text. Residue numbers were adapted to LUC1. (D) Comparison of 3D structure with LLa2 protein predicted by AlphsFold2 and LUC1 (PDB ID: 2D1S). The luciferyl-AMP analogue DLSA is drawn by the van der Waals model, and the small red circles indicate water molecules located around the substrate, which was selected from the LUC1 structure. The amino acid residues of LLa2 are red, and LUC1 are grey, blue, and green; blue and green residues indicate important residues for substrate-binding site and emission colour change, respectively.

We cloned and expressed these candidates using *E. coli* cold shock expression system. After elution through a His-tag affinity column, eluted proteins were confirmed by sodium dodecyl sulphate–polyacrylamide gel electrophoresis. However, it was difficult to purify a single-band protein due to contamination with some fragmented smaller proteins than the expected molecular weight. This could be attributed to the self-cleavage reaction. Therefore, luminescence activity was measured using the partially purified protein, and compared the luminescent activity with the expression vector product without the insert. Emission was detected, although the intensities were very weak, ranging from 1/10,000 to 1/100,000 of that of the LUC1-catalysed bioluminescence reaction (Fig. 2A). LLa2 showed the highest luminescent intensity. The maximum emission wavelength of LLa2 at pH 8.0 was around 560 nm (Fig. 2B). Since the maximum wavelengths of LUC1 and LUC2 of this species are known to be 560 and 540 nm, respectively, the emission pattern of LLa2 is similar to that of LUC1. However, the pH dependence of LLa2-catalysed bioluminescence colour was inconclusive because the luminescence intensity decreased to an undetectable level at an acidic pH. The spectra of LLp1, LLp2, and LLp3 were also difficult to measure owing to low emission intensities, even at pH 8.0.

Twelve important residues for substrate recognition and five residues for spectrally relevant sites have been detected in the LUC1 protein.^48^ These residues are mostly located within 4 Å from the luciferin substrate. Considering the protein structure predicted by AlphaFold2, three residues, 341G, 349G, and 315A were extracted, and the luminescent potential of luciferase candidates was evaluated from the perspective of enzyme–substrate interactions with these residues (Fig. 2C and D, and Supplementary Fig. S3). In the firefly luciferases discovered to date, amino acid residue 341 (with position number corresponding to the LUC1 protein) is always glycine. This is because this residue is in contact with the luciferin substrate, and even if the amino acid side chain is a methyl group, such as alanine, it is expected to have an undesirable effect on the recognition of the luciferin substrate. The serine at residue 349 enhances luminescent activity by forming hydrogen bonds that anchor the substrate in the active site through water molecules.^48^ Other amino acid substitutions without a hydroxyl group on the side chain are thought to disrupt this hydrogen bond and prevent the sufficient fixation of substrates in the enzyme. Thus, the enzymes might be not efficient for bioluminescence reactions. On the other hand, alanine at residue 315 is located more than 4 Å away from luciferin, and there is a relatively large space around this position. Therefore, the structural relaxation could allow the side chain to avoid contact with the substrate. However, compared with that of LUC1, the substrate-binding space was reduced, which could affect substrate uptake and retention as well as bioluminescent activity. The replacements of these residues in newly discovered luc-like proteins could adversely impact bioluminescence. However, optimization of these residues during evolution may have resulted in the acquisition of highly efficient luciferases in *N. cruciata*, such as LUC1 and 2.

### 3.4. Phylogenetic relationships of novel luciferase-like proteins

A phylogenetic analysis was performed for luciferases/ACSs using DmelPACS (CG6178), DmelPDGY (CG9009), and DmelACSX3 (CG11407), from *Drosophila melanogaster* as outgroups. The PACS, DmelPACS, had the PTS1 sequence and is thought to be involved in fatty acid β-oxidation at the peroxisome. Previously characterized luciferases belong to this ACS family with PTS1. On the other hand, the newly discovered luciferase-like proteins lacked the PTS1 sequence, and some showed higher sequence homology to the Pudgy protein, DmelPDGY, which functions in mitochondria.^49^

**Figure 3.**
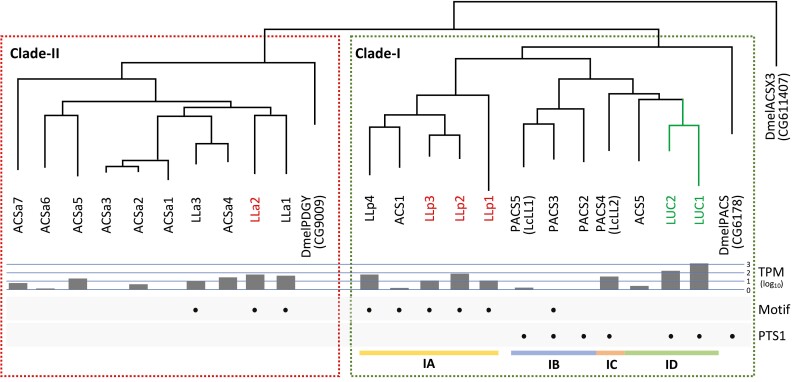
Phylogenetic tree of firefly luciferases and related ACSs. Known luciferases are shown in green letters and novel luciferase-like proteins are shown in red letters. A green box indicates Clade-I, and a red box indicates Clade-II. Subclade-IA, -IB, -IC, and -ID are indicated by yellow, blue, orange, and green bars, respectively. Gene expression levels (TPM) in lantern are shown in the logarithm. Black circles show the presence of the ‘Firefly-Luc-like’ motif (Motif) and PTS1.

The phylogenetic tree (Fig. 3 and Supplementary Fig. S4) showed that the luciferase-related ACSs could be clearly classified into two clades, Clade-I and -II. Clade-I contained the known luciferases, such as LUC1 and LUC2, with DmelPACS at the base. Therefore, the primitive character state of PTS1 possession was considered positive in this clade. This clade was further divided into two subclades, Subclade-IA and -IB-D. Subclade-IB-D included all known luciferases of the families Elateridae, Phengodidae, Rhagophthalmidae, and Lampyridae. This subclade includes PACSs and ACSs. On the other hand, all Subclade-IA members lacked the PTS1 sequence. Some of the novel luciferase-like genes, i.e. LLp1–4, belonged to this subclade, and luminescence activity was confirmed in LLp1, 2, and 3. In Clade-II, DmelPDGY was located at the base, and all members were ACSs without PST1. The remaining luciferase-like genes, LLa1–3, were included in this clade, and luminescence activity was confirmed in LLa2. These results provide the first evidence in fireflies that ACSs lacking PST1 have weak luminescence activity. Otherwise, in a non-luminescent giant mealworm, *Zophobas morio* (Coleoptera; Tenebrionidae), a luciferase-like luminescent protein without PST1 signal was isolated and cloned in the AMP-ligase gene family.^50^

LLa2 showed the highest luminescence intensity among novel luciferase-like proteins. Surprisingly, unlike other luciferases, LLa2 was derived from a common ancestor with non-peroxisomal DmelPDGY rather than with DmelPACS. The discovery of LLa2 indicates that an ancestral ACS might have been ready to acquire luminescent activity before it acquired the peroxisome targeting signal, i.e. before the divergence of PACS and PDGY. Furthermore, although there is no direct evidence, some bioluminescent genes might function in mitochondria as well as in peroxisomes, at least in *N. cruciata*. Considering the high luminescence intensity and expression level, LUC1 (and peroxisomes) is clearly important in firefly luminescence. However, the discovery of a novel luciferase-like protein that does not originate from PACS and possibly functions in mitochondria is noteworthy, providing new insights into the acquisition of luminescence ability during the evolution of firefly luciferase.

### 3.5. Comparative genome analysis of luciferase gene clusters

**Figure 4.**
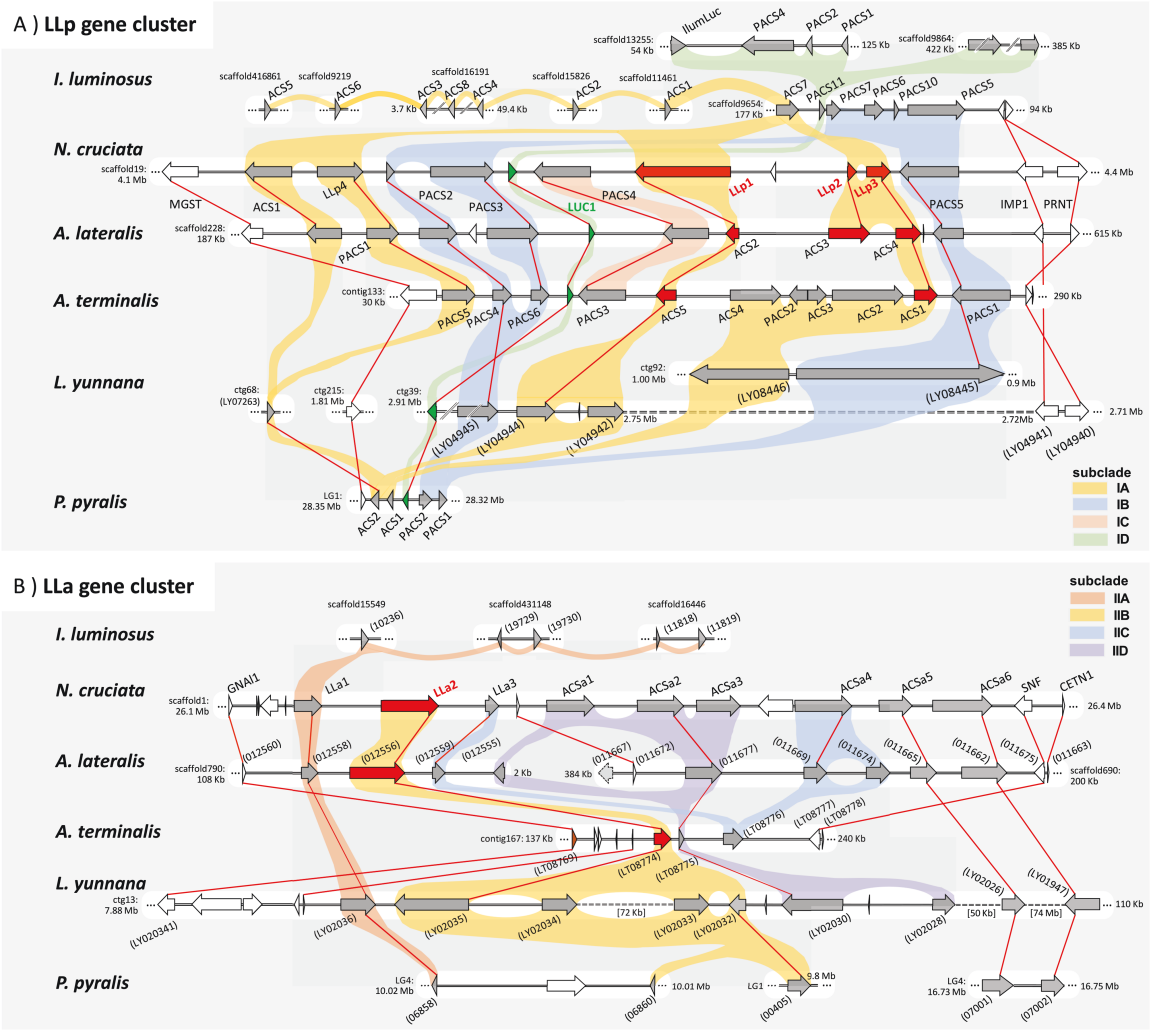
Comparison of genomic structures of luciferase gene clusters. (A) The LLp gene cluster containing *Luc1* and novel luciferase-like proteins *LLp1*, *LLp2*, and *LLp3*. (B) The LLa gene cluster containing a novel luciferase-like protein *LLa2*. Novel luciferase-like proteins and their orthologues are indicated by red arrows, *LUC1* by green arrows, and other ACSs by grey arrows. ACSs with lengths less than 300 amino acids are indicated by dotted arrows. Genes at the ends of the gene clusters are indicated by brown arrows and other genes by white arrows. Reciprocal best hits are connected by red lines, and genes belonging to the same subclade are connected by the same colour as in Supplementary Fig. S4. Numbers in parentheses indicate gene IDs instead of gene symbols.

Luciferases and related ACSs formed gene clusters in the *N. cruciata* genome (Fig. 4 and Supplementary Tables S5 and S6). We found two gene clusters, the LLp gene cluster (Fig. 4A) and the LLa gene cluster (Fig. 4B), which corresponded well to Clade-I and -II of the phylogenetic tree (Fig. 3 and Supplementary Fig. S4), respectively. We compared the genomic structures of these gene clusters to those of the other firefly genomes.

The LLp gene cluster corresponding to Clade-I contained the known firefly luciferase *LUC1* and three novel luciferase-like proteins, *NcruLLp1*, *NcruLLp2*, and *NcruLLp3*. Members of Subclade-IB-D with PTS1 have been well studied.^7,8^ Here, we focus on members of Subclade-IA, including the novel luciferase-like proteins. The phylogenetic tree indicated that the Subclade-IA members of fireflies and *I. luminosus* expanded independently in each lineage after the divergence of Lampyridae and Elateridae. The syntenic regions were highly conserved among the Luciolinae species. There was no homologue of *NcruLLp2* in *Ab. terminalis*; instead, four *NcruLLp3* homologues were detected. In *L. yunnana*, a gene cluster containing *LyunLUC1* also contained *NcruLLp1* homologues and was relatively conserved, while homologues for *NcruLLp2* and *p3* were present in another scaffold. *Photinus pyralis* had only two members in Subclade-IB, located next to *PpyrLUC1* in the genome. *Ignelater luminosus* had eight genes in this clade; however, they were scattered throughout the genome with no clear synteny with the firefly gene cluster. However, *IlumACS7* formed a gene cluster with other PACS genes, including *IlumiPACS11*, considered the common ancestral locus of lampyrid and elaterid luciferases.^7^ In addition, *IlumACS7*, along with *IlumACS1*, was the earliest gene to diverge from the other Subclade-IA members of *I. luminosus*. These results indicate that the *IlumiACS7* locus may be the common ancestral locus for all Subclade-IA members of fireflies including novel luciferases.

The LLa gene cluster corresponding to Clade-II contained a novel luciferase-like protein, *NcruLLa2*. This gene cluster showed lower conservation than that of the LLp gene cluster. Even between *N. cruciata* and *Aq. lateralis*, the gene orders were well conserved, while the copy numbers of some ACSa genes differed among species. For *Ab. terminalis*, only three ACS genes were present within the syntenic region. However, all Luciolinae species had orthologues of *NcruLLa2*. *Lamprigera yunnana* and *P. pyralis* (but not *I. luminosus*) also had multiple homologues of *NcruLLa2*. *Ignelater luminosus* did not have a clear synteny corresponding to the LLa gene cluster and only had multiple homologues for *NcruLLa1* on multiple scaffolds. This suggests that *LLa2* evolved specifically in the firefly lineage. Since the phylogenetic relationships of LLa genes were somewhat ambiguous and the degree of synteny was low, further studies are needed to unravel the evolution of non-PACS luciferases and determine when luminescence activity was acquired.

### 3.6. Chirality-related thioesterase in *N. cruciata*

Fireflies effectively produce d-luciferin from the l-form enantiomer by a chiral inversion process through the deracemization process involving three reactions: enantioselective thioesterification, epimerization, and thioester hydrolysis. Recently, the acyl-CoA thioesterase *ACOT1* of *Ab. terminalis* was identified as a strong candidate for thioesterase responsible for the thioester hydrolysis in the deracemizative d-luciferin biosynthesis.^8^ We found five acyl-CoA thioesterase genes, *NcruACOT1*–*5*, in the *N. cruciata* genome (Supplementary Table S7). Based on a phylogenetic analysis, they were classified into two groups: *NcruACOT1* and the others (Supplementary Fig. S5). Members of the latter group, *NcruACOT2*–*5*, were homologues of human *ACOT13* and formed a gene cluster in the *N. cruciata* genome. No clear expression of these genes could be observed in the lantern. On the other hand, *NcruACOT1*, an orthologue of *AterACOT1*, showed relatively high expression in the lantern (TPM = 20.7). *NcruACOT1* had high amino acid sequence similarity to *AterACOT1* (84.1% identity against an overall average of 79.6%), and the *K*_a_/*K*_s_ ratio was very low at 0.05 (*P* = 3.82e−103), suggesting that ACOT1 is functionally constrained in Luciolinae. *LcurACOT1* was an orthologue of human *ACOT9*, a mitochondrial ACOT,^51^ and had no PTS1, similar to *AterACOT1*. There is no direct evidence that ACOT1 functions in the peroxisome and further studies are needed to elucidate the site of luciferin synthesis.

### 3.7. Absorption wavelength of *N. cruciata* opsins

Two opsin genes were identified in the *N. cruciata* genome, and phylogenetic analysis confirmed that they were classified into the LW- and UV-sensitive opsin groups, respectively (Supplementary Fig. S6 and Supplementary Table S8). To directly investigate the spectral properties of these opsins, we prepared purified recombinant proteins of the opsins and measured their absorption spectra.

The absorption maxima of the UV- and LW-type opsin photopigments with retinal are shown in Supplementary Fig. S7. Fireflies have two types of retinal (retinal and 3-hydroxyretinal), and we used retinal in our experiments because opsins bound to each of the two types of retinal are known to show basically similar spectral properties.^52^ The absorption maximum of LW-type opsin photopigment in the dark state was approximately 525 nm. It is reasonable that LW-type opsin photopigment could absorb light in the yellow-green region since the light is used by *N. cruciata* for communication. On the other hand, the UV-type opsin photopigment was expected to absorb UV light; however, the measured absorption maximum was 425 nm in the blue region. The value was not consistent with the ERG response peak for *N. cruciata* (360 nm) but was comparable to those for other fireflies in the Lampyridae family (380–420 nm) although the ERG peaks do not directly reflect the absorption property of the photopigments.^14^

The K110 residue in the *Drosophila* UV-type opsin Rh3 (corresponds to G90 in bovine rhodopsin) is responsible for invertebrate UV vision.^53^ The K110 residue was conserved in the UV-type opsin of *N. cruciata*. However, amino acids on both sides of K110 were changed to methionine, with a bulky side chain (Supplementary Fig. S8). At this position, all other Lampyridae species used in this study had the same amino acid sequence of ‘MKM’ except for ‘LKM’ in *L. yunnana*. We constructed a mutant *N. cruciata* UV-type opsin in which ‘MKM’ was replaced with *Drosophila*-type ‘VKT’ and measured the absorption wavelength. As a result, a shift in the absorption maximum of the VKT mutant to the UV region (360 nm) was observed (Supplementary Fig. S9). For nocturnal fireflies, accepting visible blue light is more important than absorbing UV light, which may explain the observed mutation.

## 4. Conclusions

We sequenced the whole genome of *N. cruciata* and constructed a high-quality genome assembly of 662 Mb in length with a BUSCO completeness of 99.1% in the genome mode and 98.1% in the proteins mode. Using the detected set of 15,169 protein-coding genes, the genomic structures and genetic background of luminescence-related genes were also investigated. Four novel luc-like proteins that exhibit significant luminescent activity did not have PTS1, and one (LLa2) originated from mitochondrial PDGY, different from peroxisomal PACS, the origin of all known luciferases to date. We also found ACOT1, which might be a thioesterase involved in the conversion of l-luciferin to d-luciferin. NcruACOT1 had very high sequence similarity to AterACOT1 and was expressed in the lantern. For light reception, two types of opsins, LW- and UV-type, were identified. We found that the absorption maximum of the UV-type opsin shifted from the UV region to the blue region, and the change was attributed to the K110 mutation. These findings are very important for unravelling the evolution of bioluminescence in fireflies, and the high-quality genome assembly for *N. cruciata* will be a useful resource for future firefly research.

## Supplementary Material

dsae006_suppl_Supplementary_Figures

dsae006_suppl_Supplementary_Tables

## Accession numbers

All nucleotide sequence reads, the genome assembly (NipCru1), and the genome annotation have been deposited in the DDBJ Sequence Read Archive (BioProject accession number PRJDB13215).
