# Supplementary material for: Genome assembly of Genji firefly (Nipponoluciola cruciata) reveals novel luciferase-like luminescent proteins without peroxisome targeting signal
Source: DNA Res. 2024 Mar 18;31(2):dsae006. doi: 10.1093/dnares/dsae006 (PMC11090084; doi:10.1093/dnares/dsae006)
Supplement: dsae006_suppl_Supplementary_Figures [file dsae006_suppl_supplementary_figures.pptx]

## Slide 1
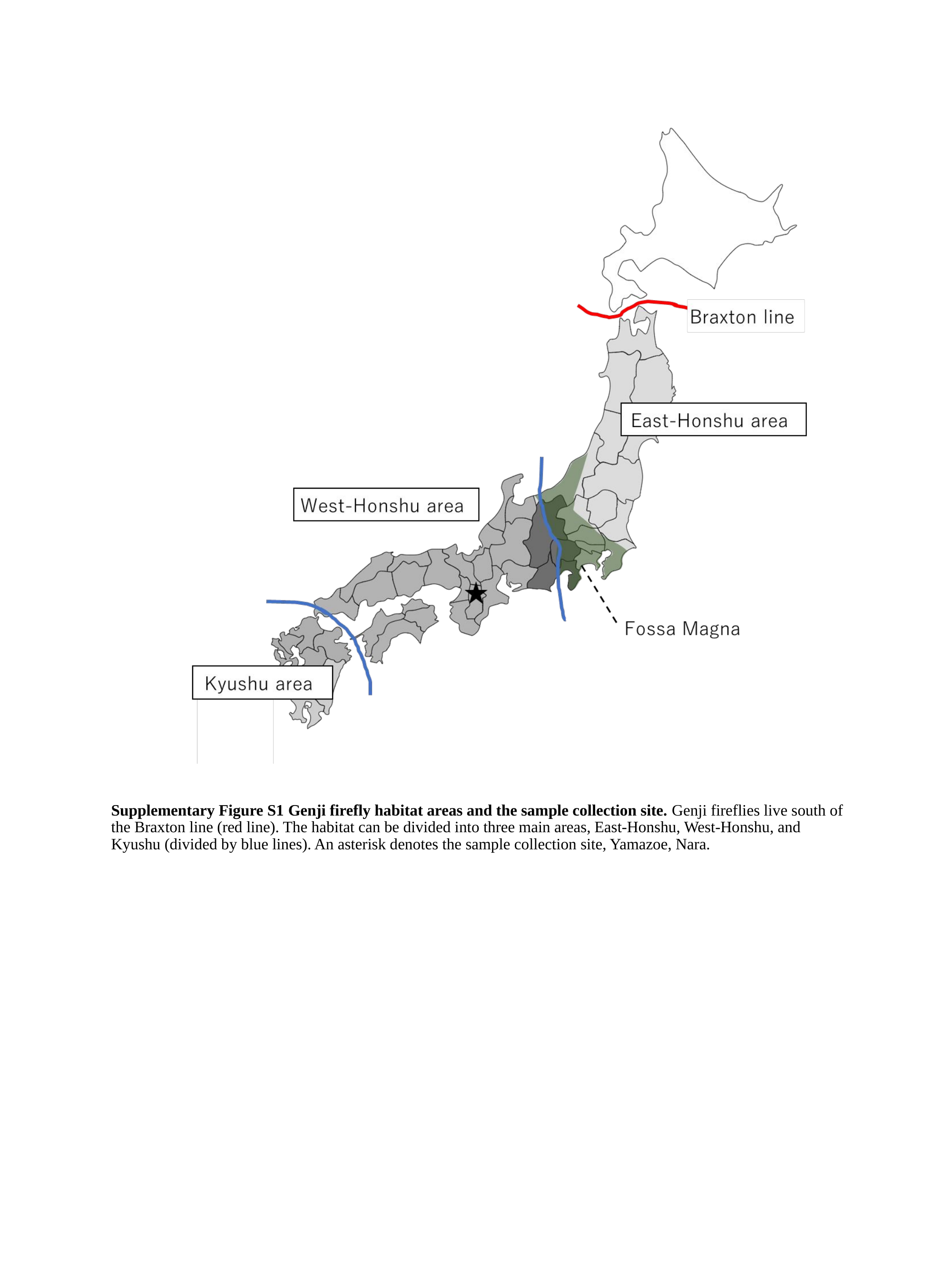

# Supplementary Figure S1 Genji firefly habitat areas and the sample collection site. Genji fireflies live south of the Braxton line (red line). The habitat can be divided into three main areas, East-Honshu, West-Honshu, and Kyushu (divided by blue lines). An asterisk denotes the sample collection site, Yamazoe, Nara.

## Slide 2
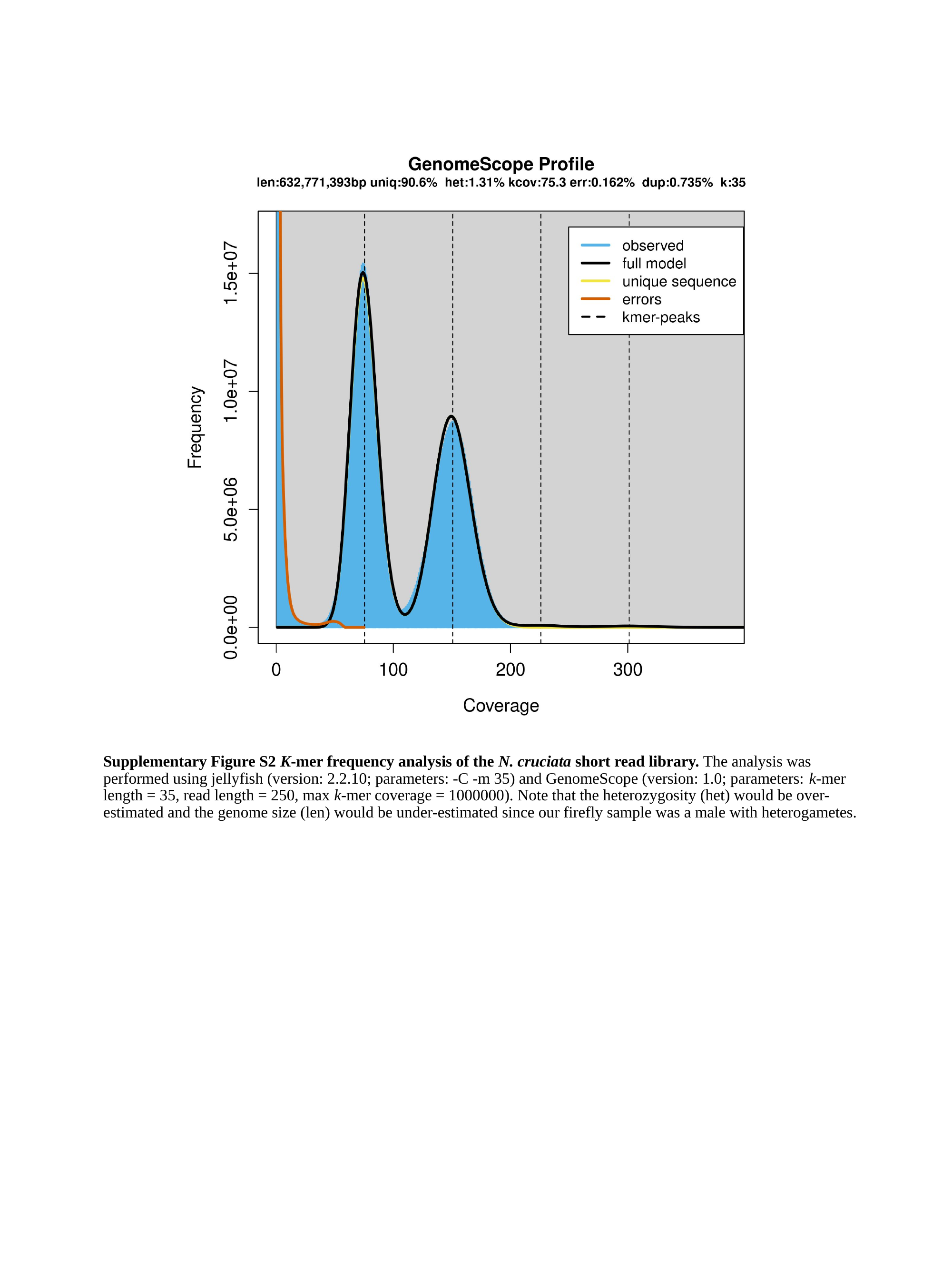

# Supplementary Figure S2 K-mer frequency analysis of the N. cruciata short read library. The analysis was performed using jellyfish (version: 2.2.10; parameters: -C -m 35) and GenomeScope (version: 1.0; parameters: k-mer length = 35, read length = 250, max k-mer coverage = 1000000). Note that the heterozygosity (het) would be over-estimated and the genome size (len) would be under-estimated since our firefly sample was a male with heterogametes.

## Slide 3
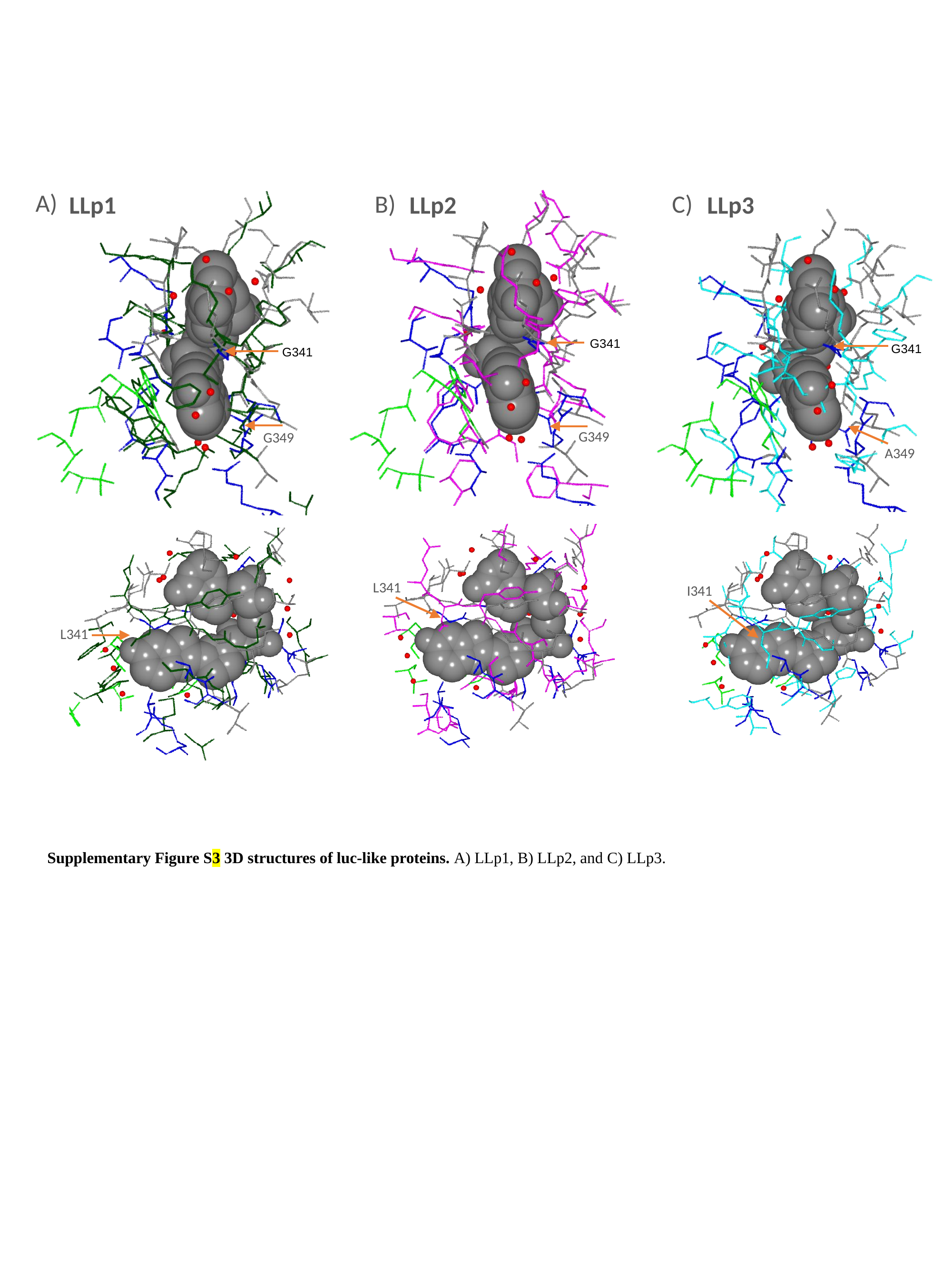

Supplementary Figure S3 3D structures of luc-like proteins. A) LLp1, B) LLp2, and C) LLp3.

## Slide 4
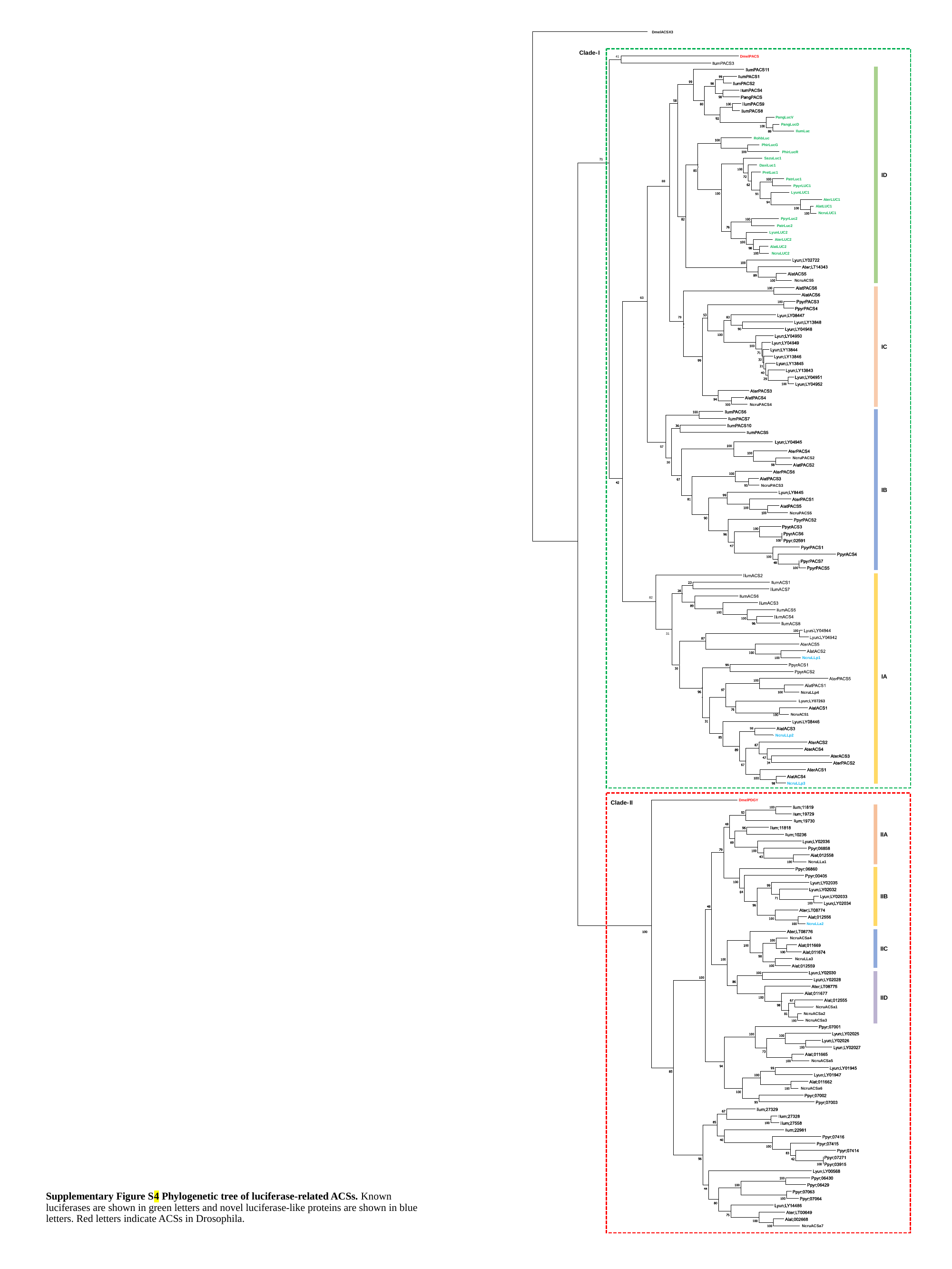

Supplementary Figure S4 Phylogenetic tree of luciferase-related ACSs. Known luciferases are shown in green letters and novel luciferase-like proteins are shown in blue letters. Red letters indicate ACSs in Drosophila.

## Slide 5
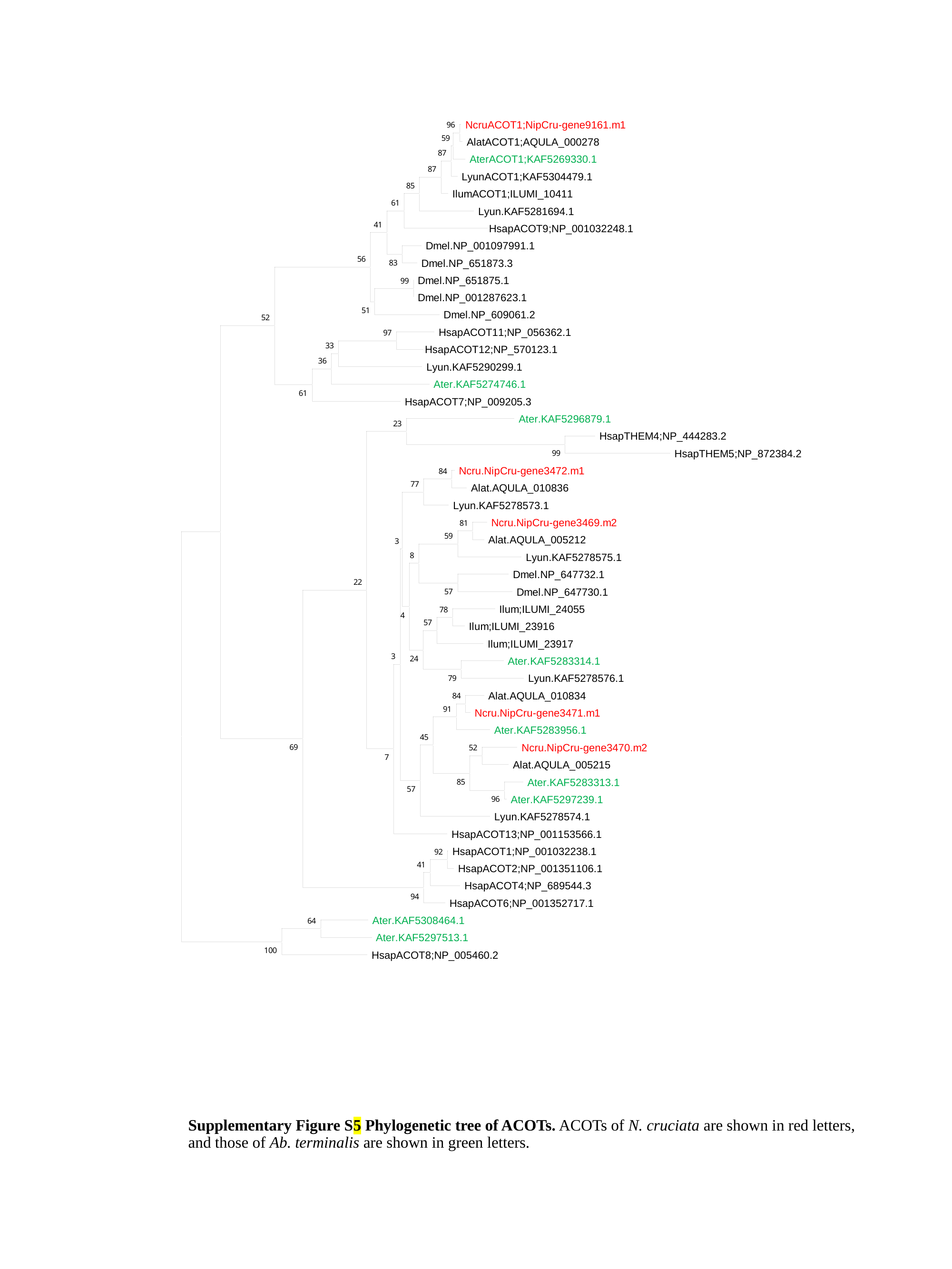

Supplementary Figure S5 Phylogenetic tree of ACOTs. ACOTs of N. cruciata are shown in red letters, and those of Ab. terminalis are shown in green letters.

## Slide 6
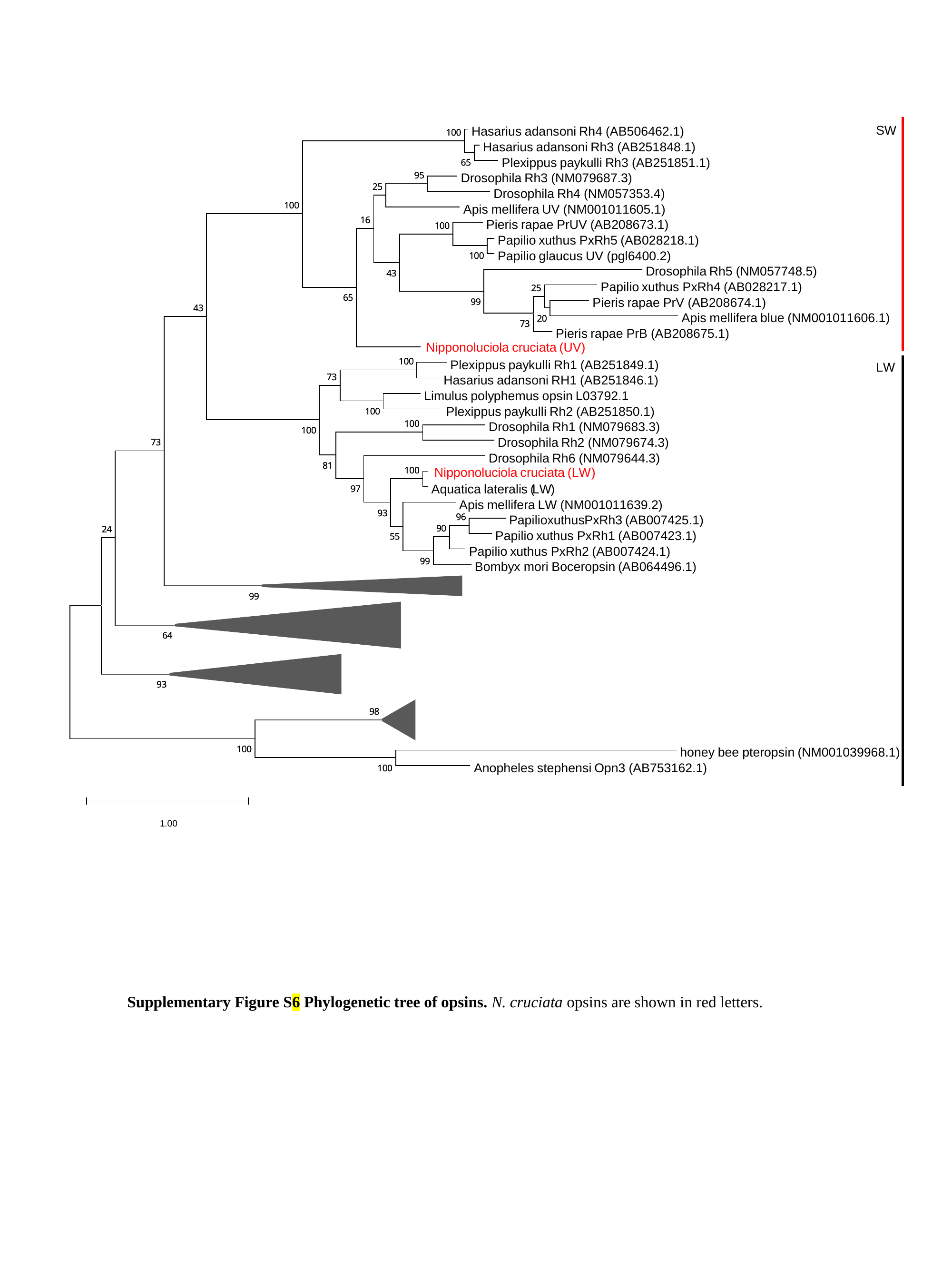

Supplementary Figure S6 Phylogenetic tree of opsins. N. cruciata opsins are shown in red letters.

## Slide 7
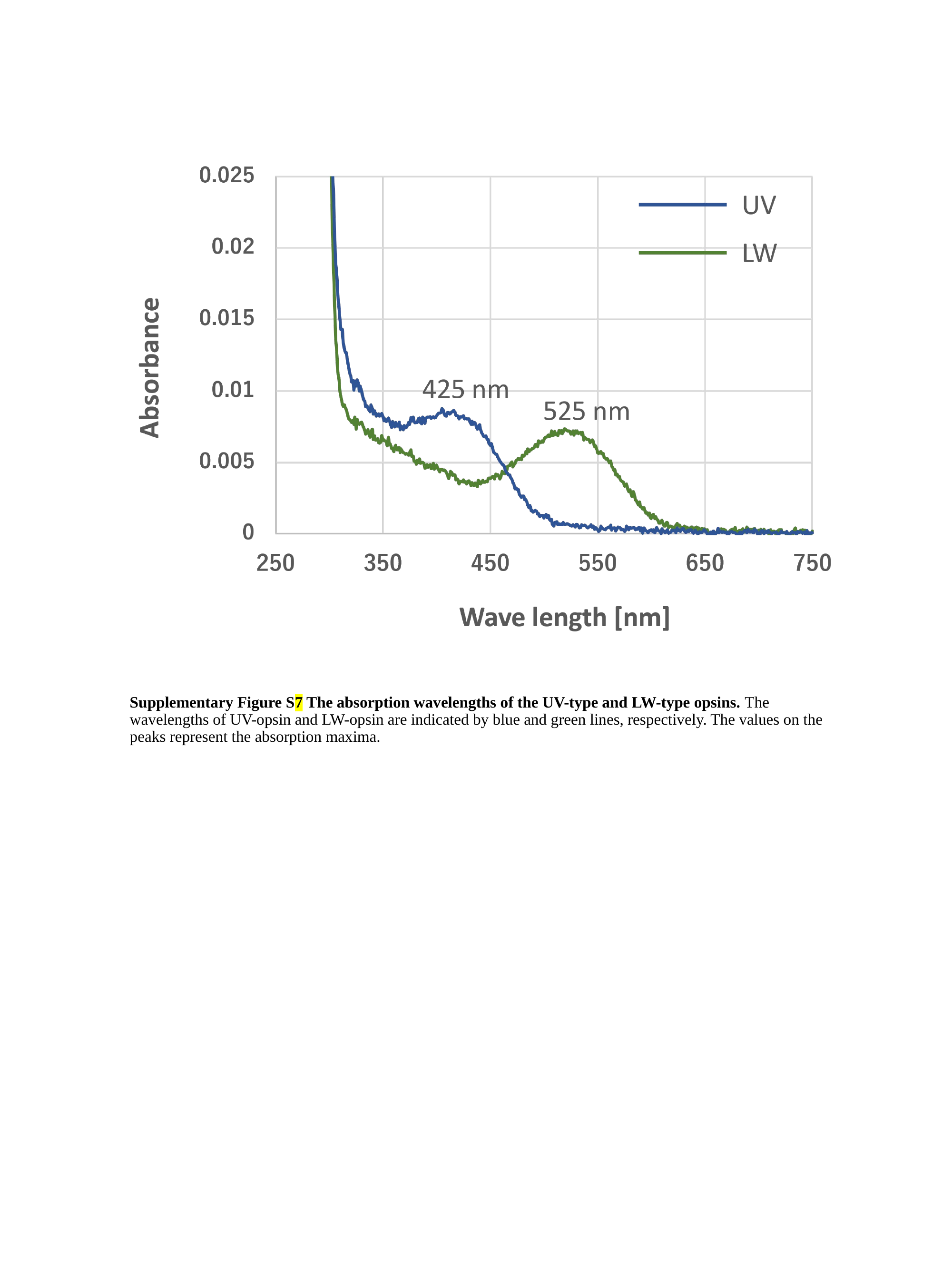

Supplementary Figure S7 The absorption wavelengths of the UV-type and LW-type opsins. The wavelengths of UV-opsin and LW-opsin are indicated by blue and green lines, respectively. The values on the peaks represent the absorption maxima.

## Slide 8
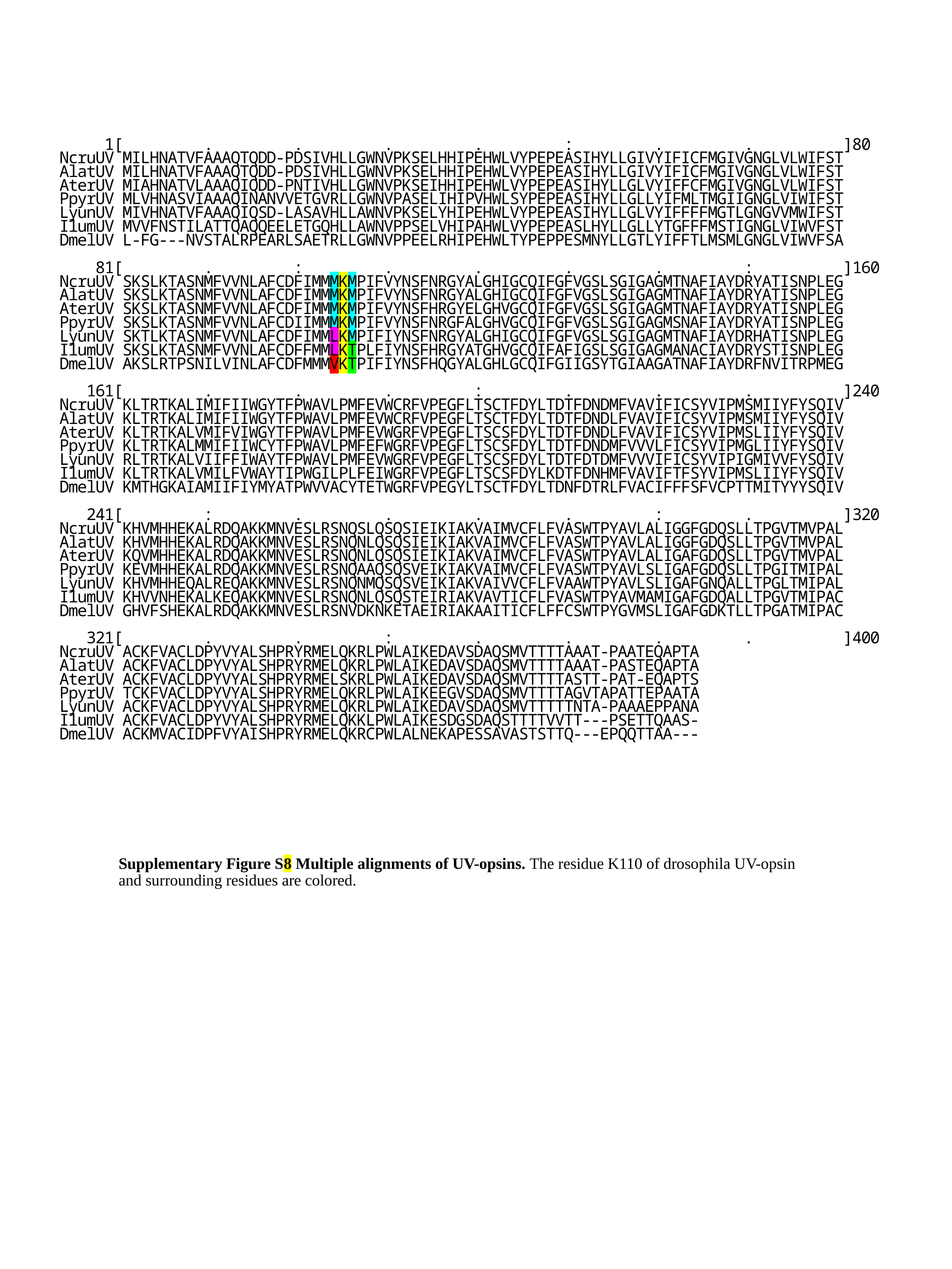

Supplementary Figure S8 Multiple alignments of UV-opsins. The residue K110 of drosophila UV-opsin and surrounding residues are colored.

## Slide 9
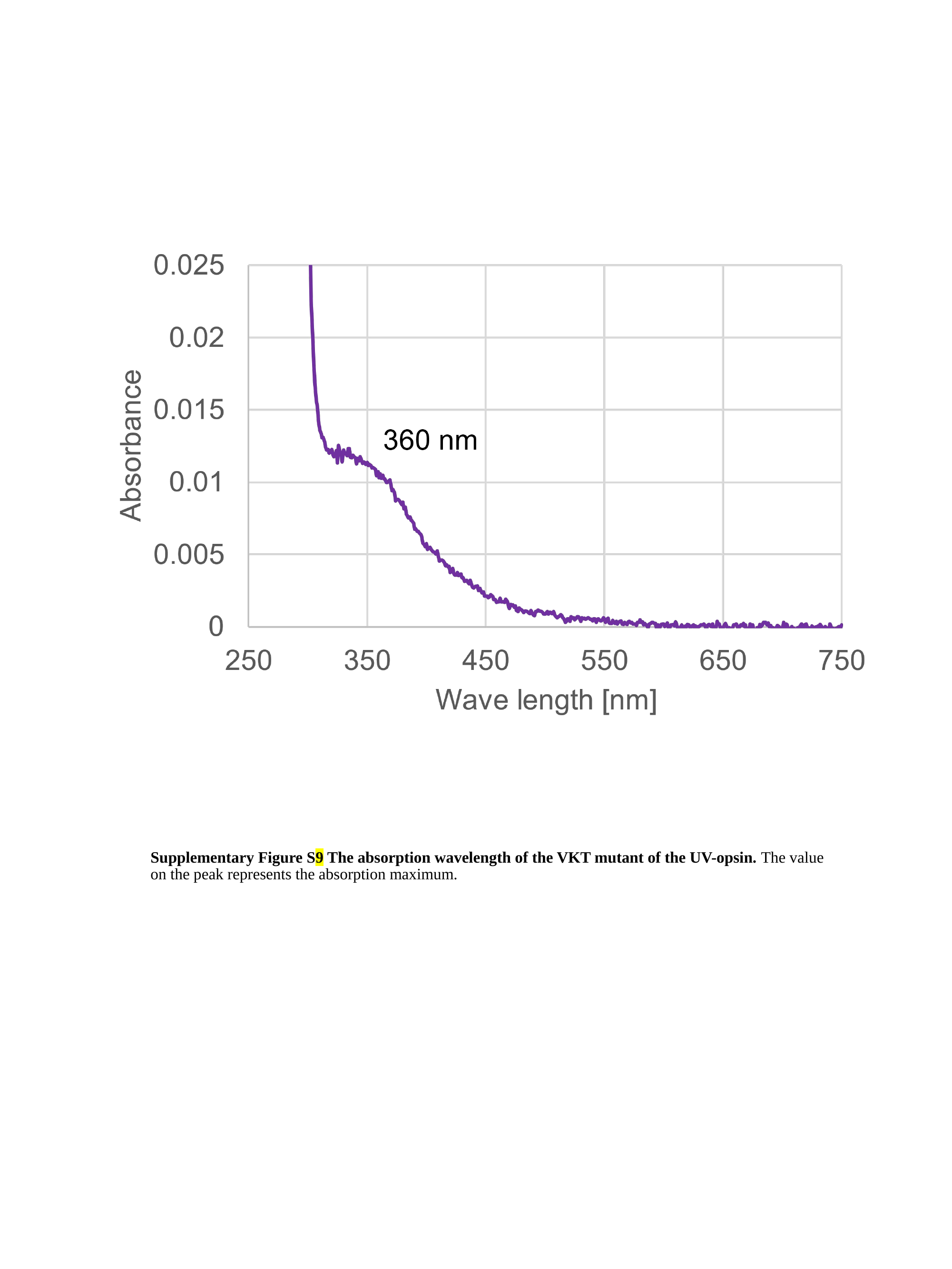

Supplementary Figure S9 The absorption wavelength of the VKT mutant of the UV-opsin. The value on the peak represents the absorption maximum.
